# Type 1 Brugada Pattern Triggered by Low-Grade Fever: Implications for Diagnosis and Risk Stratification

**DOI:** 10.3390/ijms27093900

**Published:** 2026-04-28

**Authors:** Ildikó Hamza, Lilla Végh, Veronika Sebestyén, Eszter Gulyás, Béla Juhász, Sándor Somodi, Balázs Ratku, Zsuzsanna Szűcs, Katalin Koczok, István Balogh, Zoltán Szabó, Dóra Ujvárosy

**Affiliations:** 1 Department of Emergency Medicine, Faculty of Medicine, University of Debrecen, 4032 Debrecen, Hungary; hamza.ildiko@med.unideb.hu (I.H.); vegh91@gmail.com (L.V.); sebestyen.veronika@med.unideb.hu (V.S.); gulyas.eszter@med.unideb.hu (E.G.); somodi@belklinika.com (S.S.); ratkubalazs@gmail.com (B.R.); szaboz.med@gmail.com (Z.S.); 2 Doctoral School of Health Sciences, University of Debrecen, 4032 Debrecen, Hungary; 3 Department of Pharmacology and Pharmacotherapy, Faculty of Medicine, University of Debrecen, 4032 Debrecen, Hungary; juhasz.bela@med.unideb.hu; 4 Faculty of Health Sciences, Institute of Health Studies, University of Debrecen, 4032 Debrecen, Hungary; 5 Department of Medical Genetics, Faculty of Medicine, University of Debrecen, 4032 Debrecen, Hungary; szucs.zsuzsanna@med.unideb.hu (Z.S.); koczok@med.unideb.hu (K.K.); balogh@med.unideb.hu (I.B.)

**Keywords:** Brugada, fever, coved-type T-waves, SCN5A, sudden cardiac arrest, ventricular arrhythmias

## Abstract

Brugada syndrome (BrS) is a rare but potentially life-threatening condition that may lead to sudden cardiac death. Among the causes, dysfunctions of ion channels involved in the cardiac action potential (specifically in *SCN5A* and *SCN10A* genes) are particularly significant. Among diagnosed Brugada patients, fever-induced episodes occur in 20–30% of cases. Fever worsens sodium channel dysfunction, as elevated temperature further reduces their conductance. First clinical manifestation of BrS occurs usually during a febrile episode, especially in young people. We performed a multiparametric examination in addition to genetic analysis. We treated a 19-year-old man presenting with subfebrility. During the patient’s subfebrile episodes, 12-lead ECG recordings revealed ST-segment elevations in leads V1–V3. Notably, the patient remained asymptomatic. Targeted genetic testing of *SCN5A* did not reveal any disease-causing variants as an underlying cause of the syndrome, but the temperature-inducing effect was demonstrated. The occurrence of the Brugada type 1 pattern has also been observed at subfebrile episodes, although significantly rarely. This case demonstrates that in susceptible patients, even a relatively mild elevation in body temperature can trigger ion channel dysfunctions. Timely diagnosis and follow-up are important in preserving quality of life and preventing fatal outcomes.

## 1. Introduction

Brugada syndrome (BrS) is a rare but potentially lethal cardiac ion channelopathy associated with malignant ventricular arrhythmias and sudden cardiac death in individuals with structurally normal hearts. Global prevalence varies substantially across populations; aggregated data estimate an average prevalence of approximately 0.5 per 1000 individuals (0.05%) [1]. In Asian populations, particularly in Southeast Asia—such as Thailand, the Philippines, and Japan—the reported prevalence is markedly higher, reaching 0.1–0.5% [2]. BrS typically presents in early-to-middle adulthood, most often between 30 and 50 years of age, and exhibits a pronounced male predominance, with an 8–10-fold higher prevalence in men compared with women. Registry findings show that women with BrS tend to be asymptomatic more often and are typically 6–7 years older than men at both diagnosis (49 vs. 43 years) and at the onset of the first arrhythmic event (50 vs. 43 years) [3,4]. The higher incidence of Brugada ECG pattern in adult males compared with females indicates a potential role of testosterone in ventricular repolarization [5]. Androgenic influences are believed to contribute significantly to this disparity [6]. The pathophysiological substrate of BrS is characterized by electrical heterogeneity within the right ventricular outflow tract (RVOT). This heterogeneity arises primarily from reduced inward sodium (I_Na_) and L-type calcium (I_Ca-L_) currents combined with an accentuated transient outward potassium current (I_to_) [7]. Androgens enhance I_to_ density, thereby amplifying epicardial action potential gradients and predisposing to the manifestation of the Brugada electrocardiographic pattern. In addition, androgens may downregulate sodium channel expression, further impairing conduction. Notably, I_to_ density of the right ventricular epicardium is higher in males than in females, whereas estrogen and progesterone exert stabilizing, potentially protective effects by attenuating I_to_ and preserving action potential integrity [7]. While the Brugada pattern is uncommon during childhood, its incidence increases after puberty in parallel with rising androgen levels. Observational data indicate that individuals with reduced androgen levels—such as castrated men or those with hypogonadism—exhibit a diminished prevalence of the Brugada ECG pattern, which may regress following testosterone depletion. In a study involving 48 male BrS patients and 96 age-matched controls, circulating testosterone concentrations were significantly higher in the BrS cohort (631 ± 176 ng/dL vs. 537 ± 158 ng/dL; *p* = 0.002) [6]. A clinically illustrative case has been reported in which a biologically female, female-to-male transgender individual developed a Brugada ECG pattern following the initiation of testosterone therapy, subsequently experiencing an arrhythmic event. The Brugada pattern diminished or resolved when testosterone administration was discontinued, providing strong clinical evidence that exogenous androgens can modulate the Brugada phenotype. Current studies similarly suggest that an enhanced androgenic milieu promotes conditions favorable for phenotypic expression; however, the precise underlying mechanisms remain incompletely understood.

Brugada syndrome is a complex electrophysiological disorder in which both the repolarization and depolarization hypotheses contribute to the arrhythmogenic substrate, and these mechanisms may coexist within the same patient (Table 1).

According to the repolarization hypothesis, the primary mechanism involves an imbalance of cardiac ion currents: reductions in the inward sodium current (I_Na_) and L-type calcium current (I_Ca-L_), accompanied by an increase in the transient outward potassium current (I_to_). This shift produces an epicardial repolarization abnormality in which the early “dome” of the epicardial action potential is lost, while the endocardial dome is preserved, resulting in a marked transmural voltage gradient. Such electrical heterogeneity creates a substrate that facilitates phase-2 reentry and reentry-like ventricular arrhythmias.

**Figure 1:**
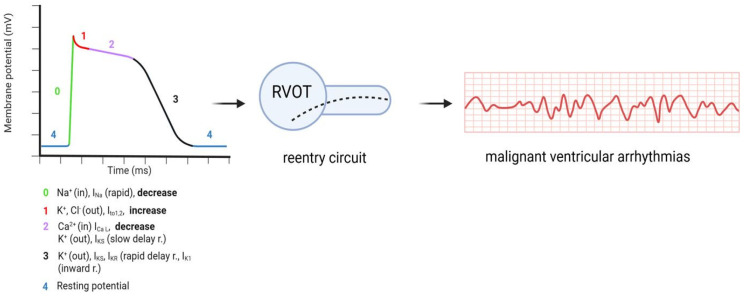
Potential alteration of monophasic action potential due to BrS. These effects may be increased in the presence of febrile state. RVOT: right ventricular outflow tract.

In contrast, the depolarization hypothesis posits that conduction delay within the right ventricular outflow tract (RVOT) is the principal driver of arrhythmogenesis (Figure 1). This mechanism is frequently associated with pathogenic variants in *SCN5A*, the gene encoding the cardiac voltage-gated sodium channel. *SCN5A* variants are associated with a broad spectrum of sodium channelopathies, including Brugada syndrome, long-QT syndrome type 3, cardiac conduction disease, and multifocal ectopic Purkinje-related premature contraction syndrome (MEPPC), highlighting the phenotypic heterogeneity associated with sodium channel dysfunction [8,9,10]. Loss-of-function mutations reduce I_Na_, leading to impaired depolarization and the development of fragmented electrograms and localized zones of slow conduction. These abnormalities alter action potential morphology, particularly in epicardial myocytes of the anterior right ventricle.

Clinical genetic testing identifies a causative molecular variant in approximately 30–35% of patients, while the remainder are classified as idiopathic. Brugada syndrome is a disease with an autosomal dominant inheritance pattern with predominantly incomplete penetrance, but autosomal recessive, X-linked and mitochondrial inheritance cases were also reported [11]. Furthermore, based on recent research results, instead of the classic monogenic inheritance pattern, the role of the so-called oligogenic inheritance is more likely in patients with Brugada syndrome, considering the altering role of genetic modifiers, copy number variations, single nucleotide polymorphisms, cis regulatory variants, micro RNA, tRNA, and mitochondrial mutations which may exacerbate or suppress the effect of the primary genetic defect [12]. The most common inherited ion channel defect implicated in the development of Brugada syndrome is a pathogenic variant in *SCN5A*, which accounts for roughly 25% of diagnosed cases. Additional genetic abnormalities occur at lower frequencies (Table 2). Overall, mutations affecting sodium channel genes (*SCN5A*, *SCN10A*) constitute about 30% of cases, calcium channel–related variants account for approximately 10%, potassium channel mutations for about 5%, and variants in other regulatory proteins for an additional 5%.

The molecular basis of fever-induced Brugada syndrome is primarily related to loss-of-function variants in the *SCN5A* gene, which encodes the cardiac voltage-gated sodium channel Nav1.5. It is the principal determinant of phase 0 of the cardiac action potential and its activity generates the fast inward sodium current (I_Na_), which is essential for maintaining an adequate conduction velocity and conduction safety factor. Pathogenic variants lead to a reduction in I_Na_ through multiple, often overlapping molecular mechanisms that become more pronounced at elevated body temperatures.

The unmasked Brugada ECG pattern during fever can be mainly caused by five major abnormalities: temperature-dependent gating alterations; conformational instability and thermosensitive protein structure; trafficking defects and proteostatic disturbances; disruption of the inward–outward ionic current balance, and post-translational as well as inflammatory modulation [13].

### 1.1. Temperature-Dependent Gating Alterations

The kinetics of the Nav1.5 channel are inherently temperature-sensitive. At elevated body temperatures, both activation and inactivation kinetics accelerate physiologically. However, in mutant channels, this may lead to a disproportionate loss of function. Characteristic abnormalities include a negative shift of the steady-state inactivation curve, accelerated inactivation time constants, reduced open-state stability, and delayed recovery from inactivation.

As a consequence, at normal resting membrane potentials, fewer channels remain available for activation. Even at temperatures of 38–39 °C, a substantial reduction in I_Na_ may occur, exceeding the myocardial conduction safety reserve [14]. Although the occurrence of the Brugada type 1 pattern at subfebrile conditions is much less frequent, it is not negligible. Based on the medical literature, subfebrility is a condition in which body temperature is persistently or transiently elevated above the normal range but does not exceed 38.0 °C, which is commonly considered the lower limit of fever [15].

### 1.2. Conformational Instability and Thermosensitive Protein Structure

Numerous missense mutations reduce the marginal structural stability of the Nav1.5 channel domains. While the quaternary conformation of the protein may remain partially preserved under normothermic conditions, fever promotes structural vulnerability: the probability of partial unfolding increases, the inactivated conformation becomes energetically favored, and the proportion of non-functional channels rises.

This temperature-sensitive instability may explain why the Brugada phenotype becomes clinically manifest exclusively during febrile state in certain individuals [14].

### 1.3. Trafficking Defects and Proteostatic Disturbances

Certain *SCN5A* variants impair intracellular protein processing and trafficking. Mutant Nav1.5 channels may exhibit increased endoplasmic reticulum retention, require chaperone-dependent stabilization, and—at elevated temperatures—undergo enhanced ubiquitination and proteasomal degradation.

The net effect is a reduction in surface channel density. Thus, fever not only alters channel kinetics but also diminishes membrane expression of Nav1.5, further reducing I_Na_ [16].

### 1.4. Disruption of Ionic Current Balance

During the early phases of the action potential, the dynamic balance between inward currents (I_Na_, I_CaL_) and outward currents (primarily I_to_) determines the morphology of the epicardial action potential. When I_Na_ is reduced, the outward currents become relatively dominant, leading to loss of the phase 2 dome in epicardial cells and the emergence of a significant transmural repolarization gradient.

Fever-induced further reduction of I_Na_ can increase this imbalance beyond a critical threshold, promoting the formation of an arrhythmogenic substrate [16].

### 1.5. Post-Translational and Inflammatory Modulation

Inflammatory mediators that excreted during fever (e.g., IL-6, TNF-α) can modulate both the expression and phosphorylation state of sodium channels. Post-translational modifications associated with oxidative stress, such as S-nitrosylation, may further reduce channel stability and conductance. Inflammation is widely recognized as physiological consequence of stress, irrespective of its duration and nature. Stress-induced systemic inflammation is characterized by increased production of pro-inflammatory cytokines (e.g., IL-6, CRP) and alterations in the type 1/type 2 cytokine balance. These changes contribute to the suppression of immunoprotective cell function, as well as to mononuclear cell aggregation and enhanced lymphocyte adhesion. Furthermore, endothelial dysfunction may arise, associated with reduced nitrogen oxide production or bioavailability, thereby promoting the development of cardiovascular pathologies, including ventricular tachycardia, atrial and ventricular fibrillation, stroke, and other acute coronary syndromes [17,18]. Although these represent secondary mechanisms, when combined with the underlying genetic background, they can lead to a clinically significant reduction in I_Na_ [19].

The clinical manifestations of Brugada syndrome (BrS) arise from electrical instability of the heart and frequently occur suddenly, without preceding warning signs. The most common symptoms include syncope, palpitations, nocturnal dyspnea, seizure-like episodes, or sudden cardiac death. Notably, up to 70% of patients remain asymptomatic. In such cases, the Brugada pattern is often detected incidentally on electrocardiography during routine evaluation or during pharmacologic provocation testing. In their analysis of ECG findings in BrS, Priori et al. reported that among 176 patients, only 90 exhibited diagnostic abnormalities on resting tracings [20]. The Brugada ECG pattern is characterized by a distinctive ST-segment elevation in the right precordial leads (V1–V3), forming the electrophysiological basis of diagnosis. Three major ECG patterns have been described, but only the type 1 pattern is considered diagnostic for Brugada syndrome (Table 3). The Brugada electrocardiographic pattern is currently classified in a simplified manner into two main categories: type 1 and non–type 1 patterns. The type 1 pattern is characterized by a ≥2 mm coved-type ST-segment elevation in leads V1–V3 with a negative T wave, and it represents the only diagnostic criterion for confirming Brugada syndrome. In contrast, type 2 and type 3 patterns are classified as non–type 1 patterns, which are not diagnostic on their own and require further evaluation to confirm the diagnosis. This simplified classification facilitates clinical decision-making and risk stratification [21]. A definitive diagnosis requires the presence of a spontaneous or provoked type 1 Brugada ECG pattern in combination with at least one compatible clinical criterion. A Brugada-like ECG pattern may also be induced by several other clinical conditions, including hypo- or hyperthermia, abnormalities in serum sodium or potassium levels, hypophosphatemia, diabetic ketoacidosis, mediastinal masses causing cardiac compression, pulmonary embolism, and myo- or pericarditis [22].

It is well established that the presence of a Brugada sign in individuals with symptoms such as palpitations or syncope is associated with a substantially higher risk of sudden cardiac death compared with asymptomatic individuals. In asymptomatic patients displaying a type 1 Brugada ECG pattern, the annual incidence of cardiac events is only approximately 1–1.5% [23]. Importantly, in patients with an existing Brugada pattern, potential arrhythmia-triggering conditions must be managed with particular vigilance, as even febrile states may precipitate malignant ventricular arrhythmias [22,23]. One of the first and most important steps in the diagnostic process is performing a 12-lead ECG, which is strongly recommended even in a low-grade fever state in patients belonging to a high-risk group, in order to detect the characteristic signs of Brugada syndrome in a timely manner.

Pharmacologic provocation testing in BrS is a diagnostic tool employed when the resting ECG does not demonstrate a definitive Brugada pattern, but clinical suspicion remains high due to factors such as family history or prior syncopal episodes. The objective of this test is to pharmacologically block cardiac sodium channels, thereby unmasking the characteristic ECG abnormality. Commonly used agents include ajmaline, flecainide, procainamide, and pilsicainide, with ajmaline being the most widely used in Europe. A recent cohort study evaluated 425 patients, comparing ajmaline and procainamide. Ajmaline yielded a positive result in 26% of cases, whereas procainamide was positive in only 4%. Overall, ajmaline use emerged as an independent strong predictor of a positive test (OR: 8.76) compared with procainamide [24]. A pharmacologic provocation test is recommended to all cardiac arrest survivors without a clear underlying cause, according to the most recent European Society of Cardiology Guidelines on ventricular arrhythmias [21]. Indeed, among these patients, 11% to 20% will develop a type 1 Brugada ECG in response to a pharmacologic provocation [25,26]. Although provocation tests are valuable, particularly in cases with intermediate clinical suspicion, their specificity is limited and they are not infallible, especially in asymptomatic or low-risk individuals [27]. A 2023 study investigated whether predictive ECG algorithms—specifically those based on r′-wave characteristics—could aid in determining whether a provocation test is warranted [28]. Fever is one of the most common provocative factors implicated in the manifestation of Brugada syndrome, as elevated body temperature further reduces the sodium current (I_Na_). This febrile trigger is diagnostically relevant because a Brugada ECG pattern may appear even in individuals without symptoms typical of the syndrome and whose electrocardiograms are normal at baseline. Only a few dozen cases have been reported in the literature in which fever either unmasked or accentuated a Brugada pattern. The prevalence of fever-induced Brugada patterns among febrile patients ranges from approximately 2% to 4% across different studies. In an Indian cohort of 525 febrile patients, type 1 Brugada patterns were identified in 11 cases (2%), all of whom were male, with a mean age of 37.7 years [29]. Another cross-sectional study from Thailand reported fever-induced Brugada patterns in 6 out of 152 febrile patients (4%). In all cases, the type 1 ECG pattern resolved once the fever subsided. Notably, two of the six patients had a first-degree relative with a history of sudden cardiac death. The authors emphasized that this prevalence (4.0%) is higher than previously recognized, especially in regions where Brugada syndrome is endemic [30].

According to a review by Viskin et al. [31], pathogenic *SCN5A* variants were identified in approximately 25% of Brugada patients presenting with fever-induced ECG patterns. Similar proportions have been reported by other groups, although some studies found lower rates, in the range of 10–15%. Overall, it can be stated that *SCN5A* mutations are confirmed in roughly one-quarter (20–30%) of fever-induced Brugada cases. The remaining cases likely reflect other genetic abnormalities or polygenic/idiopathic mechanisms. Thus, the prevalence of *SCN5A* mutations in fever-induced Brugada syndrome does not appear to be lower than in the general Brugada population. A 2024 retrospective study analyzing 263 patients with fever-induced Brugada syndrome found that individuals without *SCN5A* mutations were older on average (44.6 ± 15.7 years) and exhibited shorter PR intervals compared with those harboring *SCN5A* variants [32]. Brugada syndrome is classically considered an electrophysiological disorder rather than a primary structural heart disease; consequently, echocardiographic findings are typically normal, although subtle abnormalities may be present. Left ventricular size and function are usually preserved, and structural abnormalities are uncommon. In the right ventricle, mild dilation or wall-motion abnormalities may be observed, and some studies have described minor alterations in the right ventricular outflow tract (RVOT). Wall-motion abnormalities are most frequently localized to the RVOT region [33]. Importantly, the diagnosis of Brugada syndrome is not established by echocardiography but rather by electrocardiographic and genetic criteria. Echocardiography serves primarily to exclude other structural cardiac disorders that may mimic the Brugada ECG pattern. Mild RVOT wall-motion abnormalities may raise suspicion for early arrhythmogenic right ventricular cardiomyopathy (ARVC), but such findings are not specific to Brugada syndrome.

Management of Brugada syndrome requires a multidisciplinary approach encompassing pharmacologic therapy, implantable cardioverter defibrillator (ICD) implantation, epicardial ablation, and genetic risk assessment. Therapeutic decisions should be individualized, taking into account the patient’s clinical status and specific risk factors. Pharmacologic treatment of BrS has been the focus of considerable research and clinical investigation in recent years, comparing the efficacy and applicability of available agents. Quinidine remains the primary pharmacologic therapy, particularly in patients for whom ICD implantation is not indicated. As an I_to_ channel blocker, quinidine helps restore electrical stability in the heart. Studies have demonstrated that quinidine can reduce the incidence of ventricular arrhythmias and improve ECG patterns, inhibiting inducibility of ventricular fibrillation with approximately 90% efficacy. Long-term use has not been associated with arrhythmic events in the studied cohorts [34,35]. Isoproterenol and disopyramide are primarily used in acute settings, while their long-term use is generally not recommended. Isoproterenol, a sympathomimetic agent, increases heart rate and enhances cardiac electrical conduction. By augmenting I_Ca-L_ current and reducing the relative effect of I_to_, it stabilizes the epicardial action potential of the right ventricle, thereby decreasing the risk of ventricular fibrillation. It is considered the first-line therapy in fever-induced BrS [36]. Disopyramide, another I_to_ channel blocker, reduces action potential heterogeneity in the right ventricular epicardium and may therefore decrease the occurrence of ventricular arrhythmias [34]. Clinical studies indicate that isoproterenol is particularly effective in the management of acute arrhythmic events, including ventricular fibrillation storms, and it is shown to be effective also in children [37,38].

Research on the use of empagliflozin and ajmaline in Brugada syndrome is ongoing. Empagliflozin, an SGLT2 inhibitor, reduces cardiac workload, may shorten the QTc interval, and has the potential to decrease the risk of ventricular arrhythmias in patients with type 2 diabetes mellitus. Current studies aim to determine whether empagliflozin is effective in the management of patients with Brugada syndrome; however, results are not yet available [39]. In cases of fever-induced BrS, the aforementioned therapies should be complemented by antipyretic treatment to reduce body temperature. Evidence from research indicates that fever control improves ECG abnormalities and decreases the incidence of arrhythmic events [40].

## 2. Case Presentation

In our study, we present the case of a young male patient. On 1 February 2025, at 14:42, the National Ambulance Service (OMSZ) transported a 19-year-old Iranian male to the Emergency Department of the Clinical Center, University of Debrecen, due to fever. On-site evaluation by the ambulance team revealed stable vital signs with an elevated body temperature. The patient reported a 2–3-day history of upper respiratory symptoms, including sore throat, cough, rhinorrhea, and arthralgia. He had developed fever the previous evening. He denied chest pain or loss of consciousness. Triage assessment in the emergency department confirmed stable parameters, with a measured body temperature of 37.9 °C.

**Figure 2:**
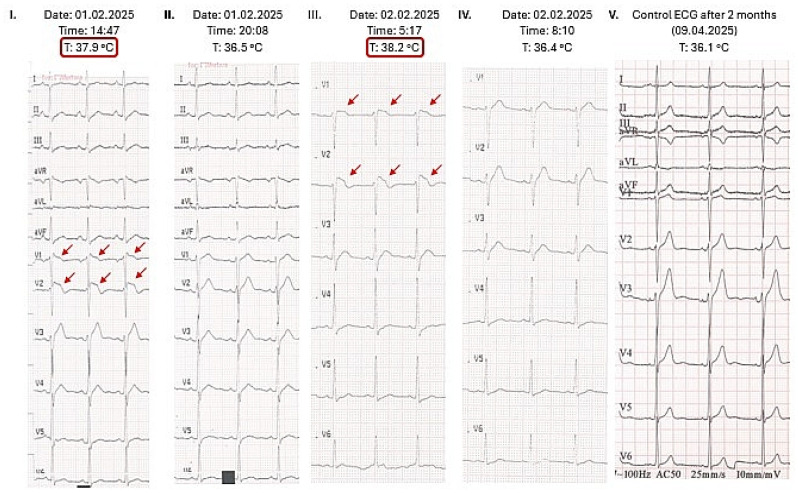
ECG alterations as a function of body temperature. Type 1 Brugada pattern can be seen on panel I and III in the case of the febrile state of our patient (marked with red). The exact time of the registration of the ECGs and the body temperatures measured at the same time are indicated at the top of every panel. T: body temperature.

During triage, a 12-lead electrocardiogram (ECG) was performed, demonstrating sinus rhythm at 102 bpm, PR interval 138 ms, QRS duration 89 ms, and QT/QTc intervals of 280/339 ms. Coved-type T-wave elevations were observed in leads V1–V2 (Figure 2). Physical examination revealed coarse vesicular breath sounds over both lungs, with no other abnormal findings. A combined rapid antigen test was performed, confirming influenza A positivity, while tests for COVID-19 antigen, influenza B, and RSV were negative. Laboratory tests showed mild elevation of C-reactive protein (CRP, 30.03 mg/L), elevated lactate dehydrogenase (LDH, 224 U/L), and creatine kinase (CK, 575 U/L) levels. CK-MB activity was 4.0%, and hypersensitive cardiac troponin T was within normal limits (5.65 ng/L). Chest radiography revealed a 5 × 2 cm area of focal decreased transparency at the mid-hilar region of the right lung; the remainder of the lung fields appeared normal. The radiographic appearance of this region was somewhat atypical. Given the ECG findings, a cardiology consultation was requested at 18:56. Based on the observed ECG changes, the absence of chest pain, and normal cardiac troponin values, acute coronary syndrome (ACS) was ruled out. The ECG abnormalities were attributed to the ongoing infection, and follow-up cardiologic evaluation was recommended. The patient remained under observation in the Emergency Department. Repeat laboratory testing demonstrated a decrease in CK (422 U/L) and LDH (202 U/L), with persistently normal troponin T levels (5.65 ng/L) (Table 4).

The patient continued to report no chest pain. He received intravenous fluid therapy (500 mL Isolyte) and 1 g paracetamol. Due to the atypical radiographic findings, a contrast-enhanced chest computer tomography (CT) was performed, which excluded the possibility of infection-induced pulmonary embolism. The CT revealed a 3.5 cm confluent, nodular consolidation in the upper lobe of the right lung. During observation, multiple 12-lead ECGs were obtained. While febrile, all recordings consistently demonstrated the characteristic coved-type ST-segment elevation in leads V1–V3 (Figure 2). Following antipyretic therapy, repeated ECGs showed resolution of these abnormalities. Prior to discharge, with the patient afebrile and in a physiological state, no repolarization abnormalities (e.g., spontaneous Brugada type 1 pattern) were observed. The patient was discharged home the following day, asymptomatic and afebrile.

Given the type 1 Brugada pattern observed on the patient’s ECG, he was scheduled for follow-up evaluation, which included repeat ECG and echocardiographic assessment. Considering the patient’s country of birth, where Brugada syndrome has an elevated prevalence, and a presumed positive family history, genetic testing was also initiated, with particular focus on the most frequently observed *SCN5A* as the gene most frequently associated with the disease. The patient presented for follow-up on 9 April 2025. He reported being asymptomatic since the initial presentation, with no chest pain, dyspnea, or loss of consciousness. ECG demonstrated sinus rhythm at 68 bpm, PR interval 107 ms, QRS duration 94 ms, QT/QTc intervals of 350/374 ms, with isoelectric ST segments and positive T waves. Echocardiography revealed no structural abnormalities, with preserved right and left ventricular systolic and diastolic function (EF: 65%, TAPSE: 23 mm).

Based on current international guidelines, a genetic test was requested for prognostic purposes and to determine the future therapeutic approach as part of the risk stratification of Brugada syndrome. For the genetic analysis, DNA was isolated from peripheral blood, and regions of genes known to be associated with the disease—including exon/intron boundaries ±20 nucleotides—were sequenced using next-generation, bidirectional sequencing on an Illumina platform. Variants were identified using the Twist Custom Panel kit (Twist Bioscience, San Francisco, CA, USA) and classified according to the ACMG/AMP 2015 guidelines [41].

Genetic analysis did not identify any pathogenic variants in the *SCN5A* gene as the most common genetic cause of BrS. According to current guidelines, no further genetic testing for Brugada syndrome is recommended. However, we examined the coding regions of 42 further genes related to cardiac disorders, such as cardiomyopathy (*ACTC1*, *ALPK3*, *BAG3*, *DES*, *DSC2*, *DSG2*, *DSP*, *FLNC*, *JUP*, *LMNA*, *MYBPC3*, *MYH7*, *MYL2*, *MYL3*, *PKP2*, *PLN*, *RBM20*, *TMEM43*, *TNNC1*, *TNNI3*, *TNNT2*, *TPM1*, *TTN*); long-QT syndrome (*CALM1*, *CALM2*, *CALM3*, *KCNH2*, *KCNQ1*, *TRDN*); and PAH—pulmonary artery hypertension (*ABCC8*, *ACVRL1*, *AQP1*, *ATP13A3*, *BMPR2*, *CAV1*, *EIF2AK4*, *ENG*, *GDF2*, *KCNK3*, *SMAD9*, *SOX17*, *TBX4*). The analysis of the examined genes yielded negative results in all cases, with no detectable variants identified across the tested genetic panel. According to the OMIM (Online Mendelian Inheritance in Men) database and Brugada et al. [42], a total of nine types of Brugada syndrome have been previously described, further associated with the *GPD1L*, *CACNA1C*, *CACNB2*, *SCN1B*, *KCNE3*, *SCN3B*, *HCN4*, *KCND3* genes that were not tested. The associations of these genes with Brugada syndrome have only recently been elucidated; therefore, their assessment is not yet included in routine genetic panel testing at our university. Moreover, only 30% of Brugada syndrome is attributed to pathogenic variants in the *SCN5A* gene [43], whereas in about 65% of the cases, the underlying cause remains unknown [44]. Whole exome sequencing can be considered as the next step to test the rest of the genes.

## 3. Discussion

In many cases, even a seemingly mild upper respiratory tract infection accompanied by low-grade fever may conceal the risk of a potentially life-threatening condition. At the same time, it may provide an opportunity for attentive and thorough healthcare providers to identify a potentially fatal disease, the timely recognition and management of which can be life-saving.

Emergency care units worldwide are under enormous pressure. The Emergency Clinic of the Clinical Centre of the University of Debrecen also provides care for hundreds of patients daily.

Despite this, it is crucial to place special emphasis and attention on patients who, based on their family history and place of birth, belong to a high-risk group for the development of arrhythmias. It is rare that, as a primary care provider, by performing serial ECG recordings on an outpatient, a Brugada pattern associated with subfebrility is revealed. Our case report highlights the importance of repeating ECG recordings during emergency care in at-risk patients when changes in body temperature occur. Based on the international findings, no other provoking factor was identified in the patient’s case.

Brugada syndrome is a dynamic ion channel disorder in which the phenotype varies in a temperature- and context-dependent manner. Temperature-dependent gating dysfunction of Nav1.5 sodium channels encoded by *SCN5A* (including slowed reactivation, enhanced inactivation, and reduced I_Na_) creates electrical instability and promotes an increased transmural repolarization gradient, thereby forming an arrhythmogenic substrate [45]. Experimental studies further demonstrate that temperature-dependent alterations in sodium channel kinetics are mutation-specific, may already be present at physiological temperatures, and can worsen with further temperature modulation [14,46]. Interestingly, Brugada-like ECG patterns occurring at lower body temperatures, even subfebrile states, have also been described in case reports and experimental models, but significantly rarely [47]. Clinical summaries, including the work of C. Antzelevitch, also emphasize that even moderate fever (~38 °C) can provoke ECG abnormalities; however, there is no universal threshold, as individual sensitivity varies [47]. Overall, clinical and mechanistic evidence indicates that the Brugada phenotype is not exclusively triggered by fever but represents a continuum of temperature-dependent ion channel dysfunction, in which both the genetic *SCN5A* background and environmental temperature jointly determine the electrophysiological expression and arrhythmic risk [47].

**Figure 3:**
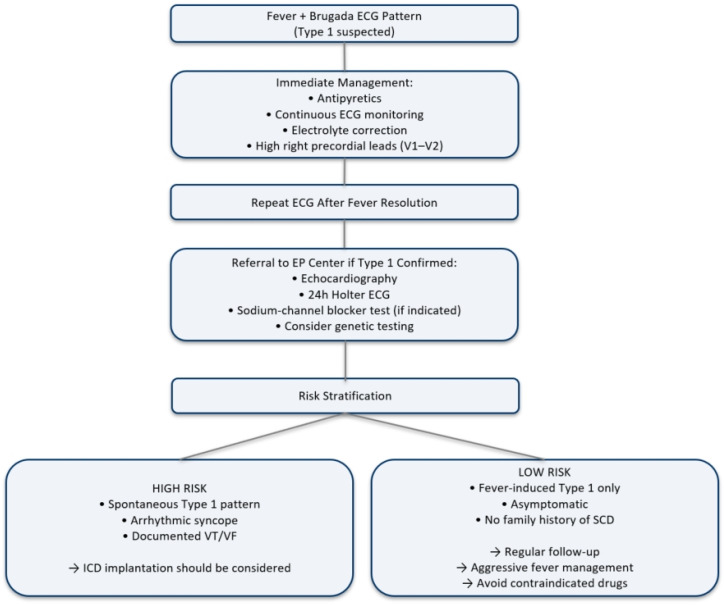
Decision algorithm and risk-stratification in case of Brugada pattern. ECG: electrocardiogram, EP: electrophysiology, VF: ventricular fibrillation, VT: ventricular tachycardia, ICD: implantable cardioverter defibrillator, SCD: sudden cardiac death.

Our observations highlight that a genetic mutation cannot be identified in all cases of Brugada syndrome; however, the fever/subfebrility-induced trigger was clearly demonstrated in our patient. Even asymptomatic individuals with Brugada syndrome may develop clinically relevant arrhythmic events during follow-up. In selected cases, prolonged rhythm monitoring using implantable loop recorders may provide additional information for risk stratification and therapeutic decision-making [48,49,50]. During follow-up, the patient remained asymptomatic, yet regular monitoring remains critically important. Implantable cardioverter defibrillator (ICD) therapy is currently the only proven intervention capable of preventing sudden cardiac death in Brugada syndrome. Its use is guided by risk stratification. In Brugada syndrome, both the Heart Rhythm Society (HRS) and the European Society of Cardiology (ESC) agree that survivors of VF/VT events or patients with syncope and spontaneous type 1 Brugada pattern on ECG are highrisk and require ICD implantation. The key difference is that HRS more often considers ICD in asymptomatic patients with spontaneous type 1 Brugada pattern ECG, whereas ESC is more conservative and generally favors observation. Intermediate-risk patients (e.g., unexplained syncope or asymptomatic spontaneous type 1 Brugada sign) require individualized decision-making, with electrophysiological stimulation (EPS) and other factors playing only a supportive role [21,51]. According to the currently accepted risk stratification system, our patient can be classified into the low-risk category, given that he remained asymptomatic throughout the observation period and had stable vital signs, that no ventricular arrhythmias were detected, and that genetic testing confirmed the absence of an *SCN5A* mutation. Moreover, the observed ECG abnormalities could be continuously detected in association with a slight increase in body temperature, whereas they were not present spontaneously and were no longer observable at normal body temperature. According to current guidelines, ICD placement is recommended in high-risk patients who have experienced cardiac arrest, documented ventricular fibrillation, or sustained ventricular tachycardia. It may also be considered in intermediate- or uncertain-risk patients, such as those with spontaneous type 1 Brugada ECG pattern accompanied by syncope. In low-risk patients—those who are asymptomatic and display only ECG abnormalities—ICD implantation is generally not advised [52]. A 2020 retrospective study examining 71 patients with fever-induced Brugada patterns found that 86% fully recovered, 3% continued to show Brugada ECG changes, and 4% died [53]. A summary publication released in 2017 suggested that in addition to the use of conventional transvenous ICDs, the implantation of a subcutaneous ICD should also be considered in patients with Brugada syndrome; however, no further supporting documentation is currently available [54]. More recent research has identified epicardial ablation as a promising new treatment option. Preliminary results in patients with electrical storm showed successful control of ventricular arrhythmias during follow-up in eight of nine individuals [55]. To support the care of affected patients, a publicly accessible website has been established, which may be freely used by both healthcare professionals and patients [53]. On this basis, drugs are classified as contraindicated or preferably avoided, and familiarity with these lists—as well as providing them to the attending physicians—is strongly recommended [56]. All of this further underscores that avoiding potentially harmful medications and ensuring effective fever control are fundamental components of adequate disease management. Based on our findings, we suggest the following diagnostic and risk stratification protocol (Figure 3) to be able to recognize these patients in an early phase to avoid potentially life-threatening conditions.

## 4. Materials and Methods

### 4.1. Study Protocol

Our study protocol was approved by the Ethics Committee of the University of Debrecen (registration number: 7346-2025 RKEB). The investigations were performed according to the Declaration of Helsinki. Our patient gave his written informed consent prior to study entry. We collected the emergency laboratory results, blood gas values, the results of the performed imaging studies, and the data about patient history, status, and epicrisis sections of the patient documentation from our electronic hospital database (UD-MED).

### 4.2. Echocardiography

A Philips HDI ATL 5000 imaging system with a 3.5 MHz transducer (Acuson Sequoia C 256, Mountain View, CA, USA) was used to perform transthoracic echocardiography. 2D, M-mode, pulsatile, and continuous—wave Doppler techniques were applied to determine the values describing the systolic and diastolic function of the left and right ventricles. In addition, the left ventricular ejection fraction, systolic right ventricular pressure, and tricuspid annular plane systolic excursion (TAPSE) were also determined.

### 4.3. Electrocardiography

ECGs were carried out four times during our examinations, according to the patient’s symptoms and after two months. ECGs were recorded at 25 mm/sec recording speed (Hewlett Packard Page Writer 200i, Hewlett-Packard Company (HP), 1939, Palo Alto, CA, USA) while the patient was in the supine position, breathing freely and not talking.

### 4.4. Laboratory Parameters

Venous blood samples were collected into Vacutainer tubes (Becton Dickinson, San Jose, CA, USA) at the Department of Emergency Medicine. Sera, citrate, and ethylenediaminetetraacetic acid (EDTA) anticoagulated plasma samples were centrifuged after 30 min at 3500× *g*, for 15 min, at +4° C. Laboratory parameters were determined from blood samples according to international standards at the Department of Laboratory Medicine of the University of Debrecen. Serum electrolyte levels, CRP, cTnT, CK, and CK-MB values, liver and kidney function parameters, and qualitative blood cell counts were measured.

### 4.5. Genetic Testing

Targeted genetic testing was performed as follows: genomic DNA was isolated from peripheral blood leukocytes. For library preparation, a custom targeted gene panel (Twist Custom Panel kit from Twist Bioscience, San Francisco, CA, USA) was used that examines the coding regions—including exon/intron boundaries ±20 nucleotides—of 43 genes related to cardiac disorders, such as cardiomyopathy (*ACTC1*, *ALPK3*, *BAG3*, *DES*, *DSC2*, *DSG2*, *DSP*, *FLNC*, *JUP*, *LMNA*, *MYBPC3*, *MYH7*, *MYL2*, *MYL3*, *PKP2*, *PLN*, *RBM20*, *SCN5A*, *TMEM43*, *TNNC1*, *TNNI3*, *TNNT2*, *TPM1*, *TTN*); long-QT syndrome (*CALM1*, *CALM2*, *CALM3*, *KCNH2*, *KCNQ1*, *TRDN*); and PAH—pulmonary artery hypertension (*ABCC8*, *ACVRL1*, *AQP1*, *ATP13A3*, *BMPR2*, *CAV1*, *EIF2AK4*, *ENG*, *GDF2*, *KCNK3*, *SMAD9*, *SOX17*, *TBX4*). Next-generation sequencing was performed on an Illumina NextSeq 2000 System in 2 × 150 bp paired-end mode, according to the manufacturer’s protocol. All detected variants having a MAF > 0.01 (minor allele frequency) in the genomAD v4 population database were filtered. The remaining variants were classified according to the ACMG standards and guidelines [41].

## 5. Conclusions

The case of our patient clearly illustrates that early recognition of fever, as well as its immediate and aggressive management, is of paramount importance in the treatment of the syndrome. In addition, it is essential to emphasize that beyond antipyretic therapy, other pharmacological effects may also influence the manifestation of the syndrome. Our case study demonstrates that even fever associated with a benign infection may elicit detectable ECG alterations. Upon identification of such findings, it is advisable to obtain a repeat recording after they have subsided and examine the evolution of these abnormalities. Our investigation indicates that this approach may carry diagnostic significance.

## Figures and Tables

**Table 1:** Pathophysiological mechanisms of Brugada syndrome. *SCN5A*: Sodium channel protein type 5, AP: action potential, RVOT: right ventricular outflow tract, I_to_: transient outward potassium current, I_Na_: rapid inward sodium current, I_Ca_: calcium current, VF: ventricular fibrillation, SCD: sudden cardiac death.

Level	Pathological Process
Genetic	*SCN5A*/Ca-channel/K-channel mutations
Cellular	↓ I_Na_/I_Ca_, ↑ I_to_ ⟶ loss of epicardial AP dome
Tissue	Conduction disorder on the epicardium of RVOT
ECG	Coved-pattern ST elevation in V1–V3
Clinical	VF, SCD among young men, can be induced by fever or certain medications

**Table 2:** Genes most commonly associated with Brugada syndrome. *SCN5A*: Sodium channel protein type 5, *CACNA1C*: Voltage-dependent L-type calcium channel subunit alpha-1C, *CACNB2β*: Voltage-dependent L-type calcium channel subunit beta-2, *KCND3*: Potassium voltage-gated channel subfamily D member 3, *SCN10A*: Nav 1.8 sodium ion channel, *SCN1B*: Sodium channel subunit beta-1, *PKP2*: plakophilin-2, *RYR2*: ryanodine receptor 2, I_Ca-L_: L-type calcium current, I_Na_: sodium current, I_to_: transient outward potassium current.

Gene	Protein/Ion Channel	Function	Effect
*SCN5A*	Sodium channel (Na_v_1.5), αI_Na_	Depolarization	↓ I_Na_
*CACNA1C*	Calcium channel subunits (Ca_v_1.2), αI_Ca_	Plateau-phase	↓ I_Ca-L_
*CACNB2b*	Calcium channel subunits (Ca_v_b2), βI_Ca_	Plateau-phase	↓ I_Ca-L_
*KCND3*	I_to_ Potassium channel subunits	Repolarization	↑ I_to_
*SCN10A*, *SCN1B*	Sodium channel accessory proteins	Conduction	↓ I_Na_
*PKP2*, *RYR2* (less often)	Desmosomal/Calcium handling proteins	Structural-electrical connection	Arrhythmias

**Table 3:** Electrocardiographic features of Brugada syndrome.

Type	Characteristic ECG Findings	Diagnostic Value of ECG	Description
**Type 1 (coved type)**	≥2 mm coved (downward-sloping) ST-segment elevation in leads V1–V2, followed by a negative T wave	Diagnostic	“Coved-type” ST-segment elevation, gradually descending into a negative T wave. Alone, this is diagnostic for Brugada syndrome.
**Type 2 (saddle-back type)**	Biphasic (“saddle-back”) ST-segment elevation, ≥2 mm initial elevation, followed by a positive or biphasic T wave	Non-diagnostic (but suggestive)	Frequently convertible to type 1 during drug provocation
**Type 3**	Similar to type 1 or type 2, but ST-segment elevation < 2 mm.	Non-diagnostic	Mild form, often indistinguishable from a normal variant

**Table 4:** Laboratory findings of our Brugada patient. Na: sodium, K: potassium, Cl: chloride, GFR: glomerular filtration rate, CK: creatine kinase, CK-MB: creatine kinase-myocardial band, AST: aspartate-aminotransferase, LDH: lactate dehydrogenase, CRP: C-reactive protein, WBC: white blood cell count.

Examination Time Points/Laboratory Parameters Assessed	1 February 2025 16:03	1 February 2025 18:33	2 February 2025 06:48
Na^+^ (mmol/L)	136	137	
K^+^ (mmol/L)	hemolyzed	3.9	
Cl^−^ (mmol/L)	102		
Glucose (mmol/L)	7.1		
Urea (mmol/L)	5.0		
Creatinine (μmol/L)	97		
GFR (mL/p/1.73 m^2^)	90		
CK (U/L)	hemolyzed	575	422
CK-MB activity (U/L)	hemolyzed	18	17
CK-MB activity (%)	not assessable	3.1	4.0
Cardiac troponin-T (ng/L)	6.06	6.08	5.65
AST (U/L)	hemolyzed	38	35
LDH (U/L)	hemolyzed	224	202
CRP (mg/L)	30.03		
WBC (G/L)	5.50		

## Data Availability

All data will be made available on request.

## Abbreviations

The following abbreviations are used in this manuscript:

BrS	Brugada syndrome
RVOT	Right ventricular outflow tract
I_Na_	Inward sodium current
I_Ca-L_	Inward L-type calcium current
I_to_	Transient outward potassium current
SCN5A	Sodium channel protein type 5
AP	Action potential
VF	Ventricular fibrillation
SCD	Sudden cardiac death
ECG	Electrocardiogram
ICD	Implantable cardioverter defibrillator
